# Pulmonary tuberculosis vs. Tindzhaka and Mafularha: A mixed methods inquiry of traditional healers’ perceptions of tuberculosis in rural South Africa

**DOI:** 10.1371/journal.pgph.0001611

**Published:** 2023-04-21

**Authors:** Carolyn M. Audet, Tshegofatso Seabi, Sizzy Ngobeni, Rebecca H. Berhanu, Ryan G. Wagner

**Affiliations:** 1 Vanderbilt Institute for Global Health, Vanderbilt University, Nashville, United States of America; 2 MRC/Wits Agincourt Research Unit, School of Public Health, Faculty of Health Sciences, University of Witwatersrand, Johannesburg, South Africa; University of Canberra, AUSTRALIA

## Abstract

Although awareness of tuberculosis (TB) is high in South Africa, delays in TB testing or treatment persist. Even those with symptoms of TB often delay testing, with one study in Mpumalanga revealing a median allopathic care-seeking delay of four weeks. We sought to understand how traditional healers perceived TB symptoms among their patients, if they treated the disease, and what (if any) illnesses they defined as being traditional may have overlapping presentation with TB in South Africa. Nineteen traditional healers completed an in-depth interview (IDIs); 133 completed a quantitative survey about their treatment practices. IDIs focused on lung diseases treated, disease causation, treatment, and prognosis. Survey questions investigated diagnosis of lung ailments, including those treated by the allopathic health system and those by traditional healers. Traditional healers reported that they could differentiate between TB and traditional illnesses, like *Tindzhaka and Mafularha*, that presented with similar symptoms. Few (7.5%) believed they could treat TB, but the majority (72.9%) believed they could successfully treat *Tindzhaka* and *Mafularha*. *Tindzhaka* and *Mafularha* are interconnected illnesses that are reportedly caused by breaking social rules around death, sex and using the belongings of someone who recently passed away. Both, if not treated, are considered fatal. While we have no definitive data, traditional healers may be contributing to delays in the diagnosis and treatment for people with active TB by incorrectly diagnosing TB as *Tindzhaka or Mafularha*. Overcoming issues of trust and compensation, while respecting different forms of knowledge, are some of the challenges we face in successfully engaging with healers.

## Introduction

In 2020, there were an estimated 328,000 new cases of tuberculosis (TB) and 61,000 deaths from TB in South Africa [1]. Early identification, referral to care, and adherence to treatment are essential to reduce TB morbidity, transmission and death. People with TB infect 5–15 close contacts annually, with household members at greatest risk [2–4].

South Africa has generally employed a passive case-finding approach to identify symptomatic patients who present at a health facility, referring those with a positive screen for testing and treatment (if appropriate) [5]. Symptom screening is done using the WHO-recommended four-symptom screen, including coughing lasting ≥ two weeks, fever, night sweats, and unplanned weight loss [6]. Very limited household contact tracing and testing is implemented as standard of care, studies of household-based active screening and testing have identified TB amongst household contacts (4.5%– 6.6%) [5, 7, 8]. However, while additional positive cases may be detected [7], in one study, less than half of those identified during community visits and referred to the clinic for a confirmatory TB test sought follow-up, with men three times less likely to attend clinic than women [5].

Despite the fact that there is a high degree of awareness about TB amongst South Africans [9], people with TB symptoms often delay in seeking care, with one study in Mpumalanga revealing a median allopathic care-seeking delay of four weeks [10]. About one-third of people with active TB who were tested >30 days after symptoms began first sought treatment from informal providers, including traditional healers [9]. There are varied reasons for this delay in care and these include not suspecting TB as a diagnosis, finding the clinic to be too far away, or not believing their symptoms were severe enough to warrant care [9, 11]. Traditional beliefs also play a role, with the majority of participants in one study reporting that TB could be cured by herbal medications delivered by traditional healers [5]. While there has not been a specific traditional diagnosis linked with TB, Tindzhaka was historically (in the 2000’s and early 10s) associated with HIV [12, 13].

Traditional healers are widespread in South Africa, with an estimated 200,000 healers across the country (compared to 46,420 biomedical doctors registered in 2019) [14, 15]. Traditional healers encompass a diverse group of individuals who perceive disease causation, diagnose, and treat illnesses using a different framework than allopathic providers, often seeing social transgressions, curses, and spiritual causes as the underlying cause of their patient’s symptoms [16, 17]. Given the pervasiveness of traditional healers, often in settings with shortages of allopathic providers, previous research has considered the role traditional healers in assisting with primary health care [18]. We sought to understand how traditional healers in Bushbuckridge, Mpumalanga perceived pulmonary TB symptoms among their patients, if they treated the disease, and what (if any) traditional illness may have overlapping symptoms with TB.

## Materials and methods

### Study design

We employed an exploratory sequential mixed methods design in this study, allowing the qualitative findings to inform the quantitative data collection [19]. This method was chosen to explore the nuanced cultural factors and perceptions associated with traditional illness, its causes and symptoms. This subsequently allowed us to develop an appropriate quantitative tool building on findings from the qualitative work using a ‘building’ approach [20]. We present the results jointly.

### Study location

**Fig 1:**
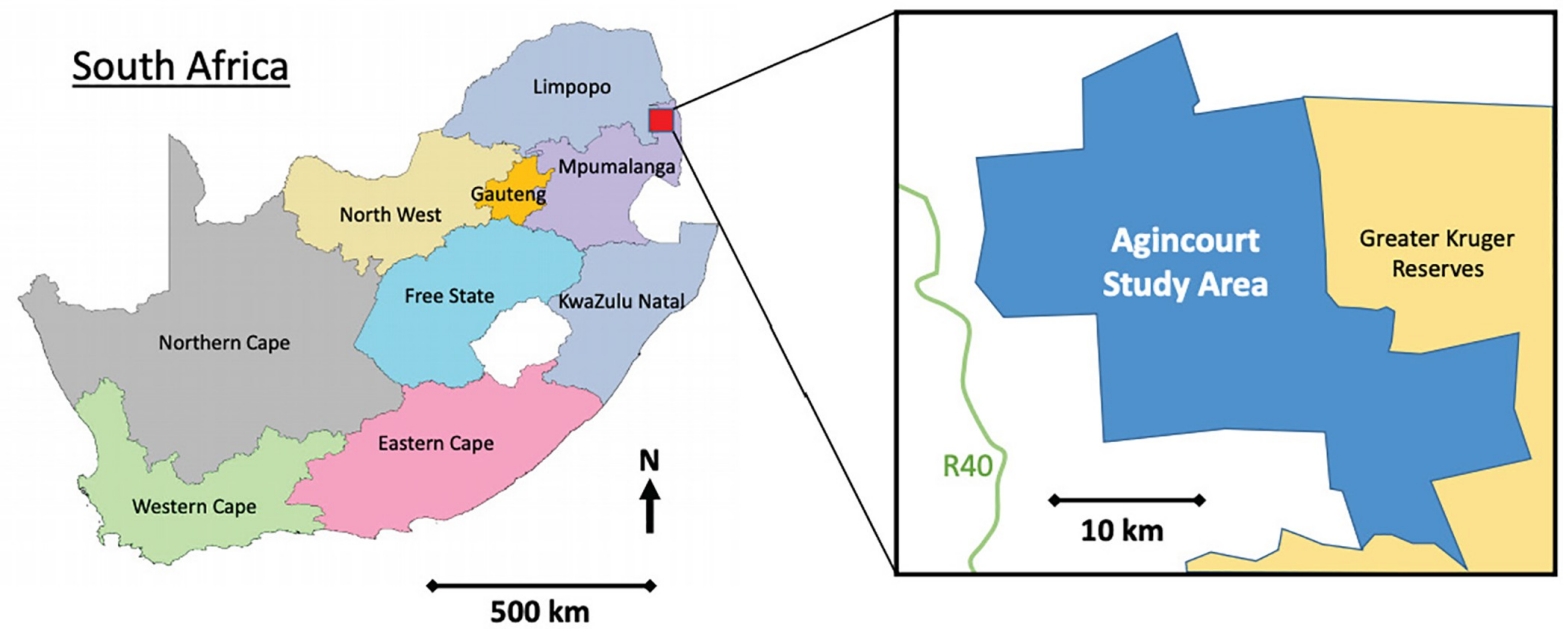
Map of Agincourt Research Area, after Wariri et al., 2017.

We conducted this study in the Bushbuckridge municipality of Mpumalanga province in South Africa, roughly 500 km northeast of Johannesburg (Fig 1: Map of Agincourt Research Area), with a population of roughly 546,215 [21]. The MRC/Wits Agincourt Research Unit (www.agincourt.co.za) manages the Agincourt Health and Socio-Demographic Surveillance Site (HDSS), which has continuously undertaken population-based health and demographic research since 1992 [22–24].

### Study population

Traditional healers registered with the Kukula traditional healer organization were randomly sampled through the random selection function in Stata (StataCorp, LP, College Station, Texas, USA) [25]. The Kukula Traditional Healer organization is the largest organized group of healers living in the Bushbuckridge area.

### Data collection

Out of a total of 280 registered healers, 169 were randomly selected to participate in the quantitative survey (allowing us to achieve a confidence level of 96% and margin of error of +/- 5%); 133 (78.7%) were identified, alive and were willing to participate. From the 133 identified, 19 healers were purposively selected to participate in in-depth interviews (IDIs) based on sex, years of practice and type of conditions treated. We collected both qualitative and quantitative data from study participants between July 2014 and August 2015.

### Qualitative data collection

IDIs were completed first to gather data regarding the illnesses healers reported treating. The IDIs, carried out by trained qualitative research staff (SN), included questions on disease causation and symptomology, healers’ preferred treatment methods and fees, conditions that healers felt they treated most effectively and their thoughts about the ability of allopathic medicine to treat these illnesses.

The IDIs were conducted in the language the traditional healer was most conversant in (mainly xiTsonga) in the location of the traditional healer’s choosing (generally, in the traditional healer’s home or place of work). Interviews lasted an average of 61 minutes. Qualitative interviews were conducted until saturation was reached. We reached data saturation after the completion of our 19^th^ interview. Types of traditional illnesses relating to lung health a healer treated, the symptoms associated with these illnesses, and it/how healers treated the conditions are specifically presented here. We also asked if the illnesses they described had an English name or were similar to any allopathic illnesses.

### Quantitative data collection

Based on findings from the IDIs, we developed a quantitative survey to determine which illness(es) healers treated, the effectiveness of treatments they provided, and fees they charged. Quantitative data collected included questions on sex, age, level of education, religious affiliation, type of traditional healing they identify with, length of training and practice, number of patients seen in the previous week and common illnesses treated, including respiratory illnesses and cost of treatment. Questions were forward and back translated into xiTsonga and administered to the traditional healer by the same fieldworker who undertook the qualitative surveys (SN). Responses were entered into RedCAP [26].

### Data analysis

#### Qualitative

Within two weeks of conducting the interview the qualitative data were translated and transcribed into English. Two research team members with considerable experience conducting qualitative research completed the thematic analysis using MAXQDA (VERBI GmbH, Berlin, Germany) software using the Framework Method [27]. This method was chosen given our interest in categorizing and comparing lung ailments (parent codes) and healer perceptions of the etiology, treatment, and anticipated outcomes associated with each disease. We used MAXQDA to generate a framework matrix to compare healer perceptions of each condition/subcode. For example, when assessing the etiology of Tindzhaka, we compared healer descriptions of causes with a focus on the primary cause(s) (social transgression, including sexual activities too soon after death in the family) and secondary causes (not completing the necessary rituals properly). We also generated codes that specifically assessed differences between conditions (e.g., symptoms of TB vs. Tindzhaka). Coding was carefully reviewed for agreement; comparison of coding for the first five interviews found 90% agreement using Cohen’s Kappa in MAXQDA®. Any incongruities in coding were resolved through consensus.

#### Quantitative

Using STATA version 17.0 (StataCorp, College Station, TX, USA), participant socio-demographic characteristics, service provision, and cost per service are presented as frequencies with percentages, and medians with interquartile ranges (IQR). Linear regressions were undertaken to examine univariate association between treating TB and key socio-demographic and traditional healer service provision-related categorical and continuous variables, with coefficients, 95% confidence intervals (95%CI) and p-values being reported. P-values < 0.05 were considered statistically significant.

### Ethical approvals

Ethical approval for this study was received from the Vanderbilt Institutional Review Board (IRB # 140646) and the University of Witwatersrand Human Research Ethics Committee (Medical) (IRB #M140547), as well as the Mpumalanga Provincial Department of Health’s Research Ethics Committee. All study participants provided written informed consent.

## Results

Healers sampled from the rural Bushbuckridge area were predominantly female (77.4%), older (59 years; IQR: 50–67]), spoke xiTsonga (n = 130; 97.3%) had long-term experience practicing traditional medicine (17 years; IQR: 9.5–30), and had low formal education levels. Ninety-two healers (69.2%) self-identified primarily as an Inyanga (herbalist) healer, while 34 (25.6%) self-identified as a Sangoma (divine treatment) healer, and 5% identified as “other” (Table 1). Participating healers who were actively treating patients at the time of the study (n = 85; 63.9%) reported seeing a median of three patients in the previous week (IQR: 1–5). Of the 19 healers who participated in in-depth interviews, 12 (63.2%) were female, with a median age of 58 (IQR: 48–67) and similar socio-demographic characteristics to the larger quantitative sample (Table 1).

**Table 1:** Socio-demographic characteristics of traditional healers who participated in quantitative and qualitative components of this work.

	Quantitative Sample (n = 133)	Qualitative Sample (n = 19)
*Female—n (%)*	103 (77.4%)	12 (63.2%)
*Age—median (IQR)*	59 (50.0–67.0)	58 (48.0–67.0)
*Type of healer*		
*Sangoma*	34 (25.6%)	2 (10.5%)
*Inynaga*	92 (69.2%)	15 (79.0%)
*Other*	7 (5.2%)	2 (10.6%)
*Completed education*		
*Primary*	52 (39.4%)	5 (26.3%)
*Secondary*	21 (15.9%)	4 (21.0%)
*Passed secondary school leaving exam*	4 (3.0%)	1 (5.3%)
*No Formal Education*	55 (41.7%)	9 (47.4%)
*Years of practicing as trad*. *healer—median (IQR)*	17 (9.5–30.0)	24 (12.0–33.0)
*Belong to an organized religion*	63 (48.1%)	13 (68.4%)

### Types of lung-diseases healers treated identified in our qualitative interviews

Healers in our qualitative interviews reported two lung-related illnesses that they could diagnosis and treat, Tindzhaka and Mafularha. None reported the ability to diagnosis or treat TB. In the process of learning more about these illnesses healers described their etiology, treatment, and prognosis, healers frequently contrasted these traditional illnesses with the TB.

### Causes of Tindzhaka and Mafularha

*Tindzhaka* and *Mafularha* are interconnected illnesses that are caused by breaking social rules around death, sex and using the belongings of someone who recently passed away. One healer explained the cause of *Tindzhaka*:

Traditional healer: *Tindzhaka is caused by taking things from the household where there is death*, *don’t ever take things to any house that there is death and bring it into your house*. *And when you are a man and you have a girlfriend in that house*, *don’t have sex with her when death has occurred*, *because if you have sex with her meanwhile there is death in her house*, *you will be attacked by Tindzhaka…*.*If you have sex with her*, *you will have Tindzhaka*, *and if you take maybe a cup or spoon and you come with it in your house*, *it is Tindzhaka and you have put your family in danger*.

Interviewer: *All the house members?*

Traditional healer: *It will infect one of the family members*, *it is not good to take things from the house that there is death; it is a taboo*. *The laws that we use in the past is making things difficult nowadays*, *especially to the children that we are having these days; you don’t have to take things from the house where there is death*, *it is a taboo and is too bad to use it*. *it is better if it’s after ten days*. (Male, 57 years)

*Mafularha* is largely seen as a sub-type of *Tindzhaka*, caused by breaking the taboo of having sex too soon after a death or a miscarriage:

*Then when there is death in the family and you find that children [younger adult family members*, *oftentimes offspring of the deceased] are having sex*, *they are not respecting that there is death in the house it causes Mafularha*. *That person will have swollen legs*, *protruding abdomen*. *Coughing and the hair becomes fluffy*, *and when the family don’t know what it is they will take hour to the hospital and the doctors will say she has TB*. *And in our tradition*, *we are able to diagnose that it is not TB and even when they try to treat her at the hospital she will not get cured*. *She will always cough*, *have fluffy hairs and swollen on the body until she dies*. (Female, 54 years)

### Symptoms and diagnosis of Tindzhaka and Mafularha

In contrast to the 7.5% of healers who treat TB, 72.9% of our survey participants believed they could successfully treat *Tindzhaka* and *Mafularha*. Healers believed they can differentiate between these traditional illnesses and TB by identifying specific symptoms, by gathering information on social taboos that may have been broken by the patient or their extended family, and by “throwing the bones” to communicate with his/her ancestors about the cause. The physical symptoms were described as a painful cough, pain in the lungs, weight loss, swollen legs and stomach, and a loss of strength over time. One healer described the physical symptoms they use to differentiate these illnesses from TB:

Traditional healer: *The coughing is not the same when a person has Tindzhaka or he has TB*, *when you test him by giving him treatment for Tindzhaka you will see that the treatment is working on him because he will be better and you have to give him another herbs that he has to inhale and he will be fine…The sputum that he will spit are not the same*, *he will cough out a sputum that is white in color and the other one will spit out the sputum that is yellow and it contains froth…The one that has froth and its yellow in color is for Tindzhaka and the one that is white is for TB*. (Male, 67 years)

Another healer provided additional information about the symptoms experienced by their patients:

*At the hospital they call it TB and in traditional way we call it Mafularha*. *When a person has TB*, *she will be coughing and spit sputum*, *that’s TB; and in our tradition when people cough without spitting sputum we call it Mafularha*. *That person will be coughing a lot without spitting and it’s Mafularha that has caused by death that was in the family… When time goes on you will see her have difficulties in breathing*, *lose appetite and have sharp pains under her breasts*. *And this shows that her lungs are have difficulties because of the air that is inside her body*. *When she starts to have difficulty in breathing*, *she will not eat well; she will start to be pale*, *weak and have pains all over her body*. (Female, 54 years)

Healers felt that the disease was quite severe and frequently, if untreated, would lead to death. One healer described the symptoms towards the end of a patient’s life.

*She will have body pains*, *headache and unable to walk*, *she will be very ill*. *She will be very ill and you have to lift her all the time when it’s worse*, *she will be coughing a lot and she also lose weight*. *She starts by having swollen body and then she will become thin and when she has lost weight*, *she will have a swollen stomach*. (Female, 74 years)

Lastly one healer described her process for identifying the specific type of traditional illness her patient had:

*I use my bones to find out*. *And when a person has Tindzhaka and you want to find out from your bones*, *you have to ask the bones by asking the name of the illness; you have to ask if it is Tindzhaka*, *or Mafularha*, *or Xidyiso* [a disease that can take many forms and is caused by the patient eating food that was bewitched]. *Because there are things that a person can be given to eat and she will be coughing like she has Tindzhaka and its not and also is not Mafularha but its Xidyiso*, *so when you are checking on the bones you have to ask if you can give her trees for Mafularha and if it’s not Mafularha the bones will refuse and when you ask if you can give her trees for Xidyiso the bones will agree it is Xidyiso*. (Female, 74 years)

### Traditional treatment of Tindzhaka and Mafularha

Treatments range from herbal remedies inhaled and/or eaten by patients, hydro massage, and the use of clothes of the person who died in treatments. None of the healers were willing to disclose the exact herbs they use, but they did describe the process. On healer provided the following details:

Traditional healer: [To treat her] *I will need clothes of a person who died and tear a piece of cloth and then I mix that small cloth with my herbs and prepare medication that I will hydro massage her using that medication*, *and I will also burn some of the hairs that I will also mix it with a piece of cloth that I have tear and I will burn it so that she inhales the smoke*. *They will continue giving her hydro massage and inhale the herbs for some days*, *and then she will be fine*. *She will be also given treatment to take*, *if she is coughing*, *she will stop coughing*. (Female, 43 years)

The same healer described how the treatment changes depending on the cause of the illness:

*If it’s a man who had sex with a woman who aborted [miscarried] a child [that caused the illness]*, *I have to ask the woman how many times has she seen her periods*, *then maybe she said I have seen my periods last month; according to our culture when you have seen your periods you have to tell the old people that you have seen your periods and they will tell you to keep everything that you are using to protect when you are menstruating because when a man is affected by Tindzhaka they will need your first menstruating diapers to treat him*. *And I will ask the man to bring his partner with all that she was using when she sees her periods after the abortion*. *Then she has to cut her nail*, *underarm and vagina hair and also hairs on the head*, *and then the man has to do it too and I will mix these things with my herbs and burn it*. *He will cover himself with a blanket to inhale the smoke of all those things that I have burned*. *After that I will tell them to go home and have sex for seven days whether the wife is on her periods or not but they have to*, *and after that he will be cured*. *If they don’t have sex for seven days*, *the man will die*. (Female, 43 years)

Traditional treatments can also take the form of razor cutting to let out the blood of a patient experiencing symptoms. Healers use razors to make incisions on the patient’s chest, back, arms, and genitals. Frequently herbs are rubbed into the incisions.

*When treating Tindzhaka*, *we are using hydro massage and also herbs that you will smoke by your mouth using a rite and also treatment that you have to take*. *Then I will also cut her with a razor*, *and lot of blood will be coming out and the blood will be black in color*. *After that she will continue taking the treatment to inhale and some are soaking and some are boiled*. (Female, 74 years)

Several of the healers noted that they preferred that patients stay with them during treatment to ensure compliance with medications. Among those who believed they could treat these traditional ailments; none were concerned that they would fail to successfully heal the patient or were concerned about becoming infected themselves.

### Survey results

Based on our qualitative results, we included three lung-related ailments in the quantitative survey: Tindzhaka, Mafularha, and TB.

Few traditional healers (n = 10; 7.5%) reported that they could diagnose and treat TB, but most believed they could effectively treat Tindzhaka and/or Mafularha (n = 97; 72.9%). The reported ability to treat TB was found to be significantly associated with healers actively treating patients and those who also reported treating *Tindzhaka/Mafularha*. There was no association between sex, age, or type of healer and treatment of TB (Table 2).

**Table 2:** Linear regressions showing univariate associations between treating TB and sociodemographic and provision-related variables.

Variable of Interest	Reports not treating TB (n = 123)	Reports treating TB (n = 10)	coeff (95%CI)	p-value
*Sex (Female)*	95 (77.2%)	8 (80.0%)	0.01 (-0.10–0.12)	0.842
*Age—median (IQR)*	59.0 (50.5–67.5)	53.5 (50.0–64.0)	-0.001 (-0.004–0.003)	0.667
*Type of healer*				
*Sangoma*	32 (26.0%)	2 (20%)	0	
*Inynaga*	84 (68.3%)	8 (80%)	0.028 (-0.078–0.133)	0.599
*Other*	7 (5.7%)	0	-0.058 (-0.277–0.159)	0.595
*Completed education*				
*Primary School*	47 (38.5%)	5 (50%)	0	
*Secondary School*	19 (15.6%)	2 (20%)	-0.001 (-0.137–0.135)	0.989
*Passed secondary school leaving exam*	3 (2.5%)	1 (10%)	0.154 (-0.118–0.426)	0.266
*None*	53 (43.4%)	2 (20%)	-0.060 (-0.161–0.042)	0.246
*Years of practicing as traditional healer—median (IQR)*	17 (10–30)	3 (16–28)	-0.001 (-0.004–0.002)	0.635
*Belong to an organized religion*	58 (47.9%)	5 (50%)	0.005 (-0.098–0.086)	0.901
*Months of training as traditional healer—median (IQR)*	10 (10–24)	18 (9–24)	-0.001 (-0.004–0.002)	0.549
*Actively treating patients in last month*	73 (61.3%)	10 (100%)	0.115 (0.046–0.183)	**0.001** *
*Number of patients treated in past week—median (IQR)*	1 (0–4)	2 (0–3)	-0.009 (-0.022–0.003)	0.131
*Reports treating/curing patients with Tindzhaka/Mafulhara*	87 (70.7%)	10 (100%)	0.293 (0.005–0.579)	**0.046** *

* Denotes significance

The few healers who did treat TB charged R700 (1 South African Rand (R) = 17.07 USD in 2022) with charges ranging from R400-1000 for treatment. This was less than they charged for *Tindzhaka* or *Mafularha*, which cost patients on average 1000 Rand (59 USD in 2022) to cure the disease.

## Discussion

While only 7.5% of traditional healers reported intentionally treating TB, 73% reported treating illnesses with symptoms strongly suggestive of active pulmonary TB disease, possibly leading to delays in allopathic care for TB, thereby increasing risk to close contacts of the infected individual and potentially leading to adverse outcomes, including death. Of concern, those who are actively treating patients and those who reported treating *Tindzhaka* and*/*or *Mafulahra* were significantly more likely to report treating TB, suggesting potential overlap and misdiagnosis among active traditional healers. Whilst not conclusive, further research confirming the TB status of individuals diagnosed with *Tindzhaka* and/or *Mafhulara* is urgently warranted.

Identifying barriers to identifying and diagnosing cases of TB, particularly those found in the community, should be a priority. Patients have expressed the belief that herbal remedies could cure TB, leading to delayed and/or abandoned care.] While healers continue to advertise cures and provide treatment, this barrier to early screening and treatment remains a public health challenge.

Ten to 15 years ago, *Tindzhaka* was likened more to HIV than to TB. In previous studies published in 2007 and 2011 participants highlighted the parallels between *Tindzhaka* and HIV, suggesting that AIDS was just the English word for an old traditional disease [12, 13]. Two additional studies also highlighted the association made by traditional healers between *Tindzhaka* and HIV [13, 17]. In all these studies, participants reported that a diagnosis of *Tindzhaka* eliminated any stigma associated with the symptoms of an HIV diagnosis. Furthermore, participants from these studies believed that the traditional illness could be successfully treated [12, 13, 17]. A diagnosis of TB is still strongly linked with HIV in the minds of many South Africans [28]. Thus, it is likely a patient would prefer a diagnosis of *Tindzhaka* or *Mafularha* to one of TB (or HIV). With the successful scaling up of HIV care and treatment in South Africa, it appears that the traditional diagnosis has shifted from a relationship with HIV to that of TB.

The most pressing question is what proportion, if any, of patients diagnosed with *Tindzhaka* or *Mafularha* have active TB? Although there are reports of patients with *Tindzhaka* eventually being diagnosed with TB [29], to our knowledge, no rigorous study has satisfactorily addressed this question. It is essential to partner with healers to screen clients and community members for active TB.

Traditional healers have partnered with allopathic healthcare providers to identify people living with a number of conditions including HIV [30–32], malaria [33], mental health disorders [34] with some success because, in part, healers and allopathic healthcare providers agreed upon the diagnosis (e.g., healers believed the patient had a disease better treated by biomedicine). Collaborations with healers to increase referrals for TB testing and treatment have been met with mixed success [35–37]. In the most recent study from an Ethiopian pastoralist community, it was found that traditional healers, when given adequate training and support (comprising 1 week of in-person training with demonstration; meetings with district TB prevention and care coordinators and health extensions workers) were able to screen and refer individuals with suspected active TB. That said, early work from South Africa were not as successful, and addressing the diagnosis gap and creating a platform for convergent understanding [29] will be essential to overcoming this challenge.

### Limitations

This study was carried out in rural South Africa by an experienced team of researchers combining the strengths of both quantitative and qualitative research to identify overlapping illnesses through quantitative methods and then more deeply interrogating these similarities (and differences) through qualitative interviews. The results presented are the views and findings of healers registered with a specific traditional healer organization and working in Bushbuckridge. As such, the quantitative results of this work may not be generalizable to healers not registered with Kukula or healers working in the rest of rural South Africa. Furthermore, discussions on types of diseases treated by traditional healers, types of treatment provided and costs of treatment can be sensitive topics and responses to this line of questioning may be influenced by perceived social preference. Efforts made to mitigate this risk included developing rapport with the healer and reinforcing the fact that these questions were being asked as part of ongoing research and would be kept strictly confidential. That said, some risk of social preference bias does remain. It is worth noting that this study was undertaken prior to the COVID-19 pandemic. Understanding how the healers’ views and practices have (or have not) changed in light of the COVID-19 pandemic through future research would likely be beneficial. Finally, the majority of the Bushbuckridge population and the traditional healers interviewed are xiTsonga-speaking and identify as Shangaan; as such, the traditional illnesses discussed, specifically *Tindzhaka* and *Mafularha*, may belong to this specific culture. Further research is warranted to understand whether other South and sub-Saharan African cultures have similar traditional illnesses that present comparably to *Tindzhaka* and *Mafularha*.

## Conclusions

Most traditional healers do not report treating TB; however, in treating traditional illnesses with similar symptoms to TB, such as *Tindzhaka*, traditional healers may be unintentionally treating people with TB. This could lead to delayed presentation at allopathic health facilities and likely poorer patient outcomes and increased community spread. Engaging traditional healers to screen and refer individuals with TB symptoms to allopathic health facilities will aid in the early identification and treatment of people with active TB. Working together with traditional healers through innovative collaborations may provide one way to further mitigate that TB burden.
